# Integration of TE Induces Cancer Specific Alternative Splicing Events

**DOI:** 10.3390/ijms231810918

**Published:** 2022-09-18

**Authors:** Woo Ryung Kim, Eun Gyung Park, Yun Ju Lee, Woo Hyeon Bae, Du Hyeong Lee, Heui-Soo Kim

**Affiliations:** 1 Department of Integrated Biological Sciences, Pusan National University, Busan 46241, Korea; 2 Institute of Systems Biology, Pusan National University, Busan 46241, Korea; 3 Department of Biological Sciences, College of Natural Sciences, Pusan National University, Busan 46241, Korea

**Keywords:** alternative splicing, TE, miRNA derived from TE, cancer

## Abstract

Alternative splicing of messenger RNA (mRNA) precursors contributes to genetic diversity by generating structurally and functionally distinct transcripts. In a disease state, alternative splicing promotes incidence and development of several cancer types through regulation of cancer-related biological processes. Transposable elements (TEs), having the genetic ability to jump to other regions of the genome, can bring about alternative splicing events in cancer. TEs can integrate into the genome, mostly in the intronic regions, and induce cancer-specific alternative splicing by adjusting various mechanisms, such as exonization, providing splicing donor/acceptor sites, alternative regulatory sequences or stop codons, and driving exon disruption or epigenetic regulation. Moreover, TEs can produce microRNAs (miRNAs) that control the proportion of transcripts by repressing translation or stimulating the degradation of transcripts at the post-transcriptional level. Notably, TE insertion creates a cancer-friendly environment by controlling the overall process of gene expression before and after transcription in cancer cells. This review emphasizes the correlative interaction between alternative splicing by TE integration and cancer-associated biological processes, suggesting a macroscopic mechanism controlling alternative splicing by TE insertion in cancer.

## 1. Introduction

The finding that more than 90% of the average pre-mRNA sequence is removed as introns in the nucleus, and only approximately 10% of the remaining pre-mRNA is combined as exonic sequences, brings forth a fundamental principle of biology, known as RNA splicing [1,2]. Alternative splicing is a regulatory process of gene expression that imparts macromolecular and cellular complexity to higher eukaryotic organisms by allowing the production of two or more variant mRNAs from a single gene. The exons of primary transcripts are spliced into structurally and functionally distinct mRNAs that have distinct arrangements by alternative splicing [3]. That is, the combination of exons is alternatively determined as the cell decides whether to eliminate a part of the pre-mRNA or include a specific part of the mature mRNA [4]. These processes are orchestrated by the spliceosome, a dynamic and powerful macromolecular machinery complex, in a synergistic and antistatic manner [5,6]. A change in alternative splicing could play a role in the occurrence of human diseases by adjusting processes including exon skipping, intron retention, and the choice of alternative splicing sites [7,8]. In particular, protein isoforms generated by dysregulated alternative splicing, known to be the hallmark of cancer, have a close relationship with cancer development and are the subject of therapeutic interventions in numerous cancers [9,10].

The insertion of TEs is expected to be the origin of alternative splicing including, exon shuffling, and constitutively spliced exons [11]. TEs are DNA sequences that can mutate the host genomes by changing their locations and facilitating chromosomal rearrangements via homologous recombination. According to the authors of [2,12] TEs, which consist of almost half of the mammalian genomes, can be classified into two major classes distinguished by their transposition mechanisms: DNA transposon and retrotransposon [13]. DNA transposons are generally extinct in higher eukaryotes and are activated through a ‘cut-and-paste’ mechanism which depends on transposase for catalyzing excision and insertion [14]. In contrast, retrotransposons change their position via a ‘copy-and-paste’ mechanism using RNA intermediates and are reverse transcribed into complementary DNA (cDNA) by reverse transcriptase while retaining the template at its original locus [15]. Retrotransposons can also be classified into two subclasses based on the presence of a long terminal repeat (LTR): LTR retrotransposons, including endogenous retrovirus (ERV) and non-LTR retrotransposons including long-interspersed elements (LINEs) and short-interspersed elements (SINEs), such as Alu and SVA elements [16,17]. Retrotransposons are subdivided into autonomous and non-autonomous retrotransposons [18]. ERVs and LINEs are autonomous retrotransposons that encode reverse transcriptase for insertion into another region of the genome [19]. In contrast, SINEs are non-autonomous retrotransposons that cannot move to other regions by themselves because of the absence of reverse transcriptase in their sequences; they require LINEs for their transposition [20].

Integration of TE into the host genome can disrupt gene function or alter gene expression by increasing alternative splicing mechanisms [21,22]. Furthermore, TEs can generate miRNAs, also called miRNAs derived from TEs (MDTE), which are 20–25 nucleotide non-coding RNA that bind to the 3′ untranslated region (UTR) of the target mRNA [23,24]. At the post-transcriptional level, miRNAs can function as vital regulatory factors by controlling the expression of specific transcripts through repressive processes, including translational suppression and initiation of mRNA degradation [25,26]. Based on the literature, it has been revealed that biological regulatory actions induced by TE insertion are significantly related to cancer development [27,28]. This review focuses on alternative splicing and TEs that have a profound effect on the onset and development of cancers through the tuning of regulatory correlations. In addition, the tumorigenic mechanisms of TE insertion and induced alternative splicing are summarized. Simultaneously, a cancer regulatory model of the interaction among TEs, alternative splicing, and miRNAs originating from TEs is presented.

## 2. The Intimate Connection between Alternative Splicing and Cancer

**Figure 1:**
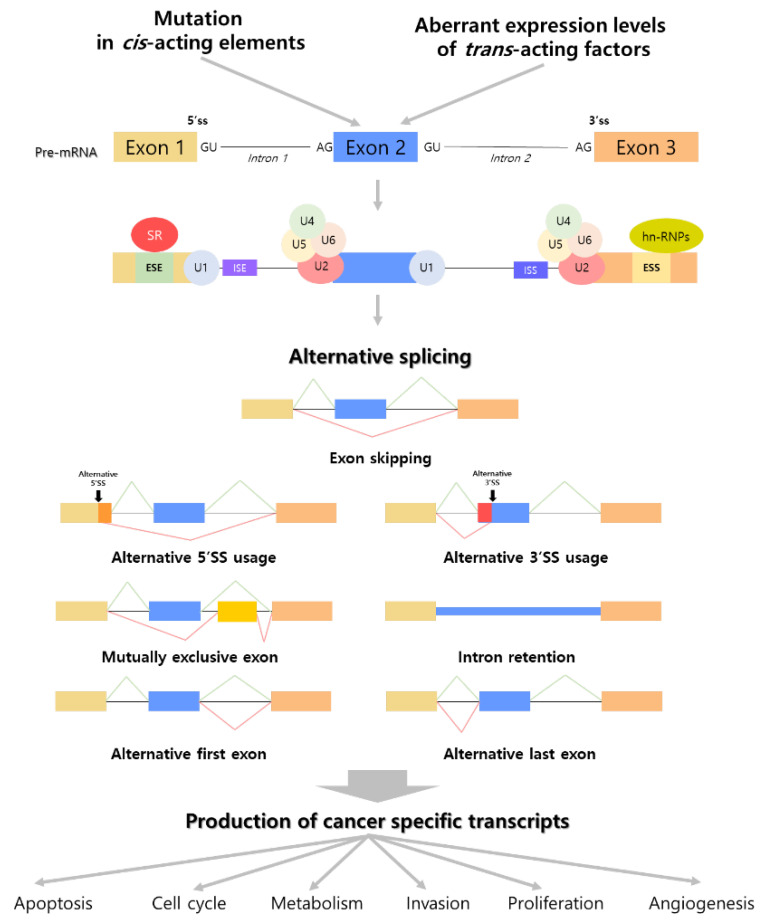
Effects of alternative splicing on biological processes related to cancer progression through the generation of cancer-specific transcripts. Alternative splicing events in cancer are activated by mutations in cis-acting elements and aberrant expression levels of trans-acting factors. The initiation of alternative splicing is stimulated by aggregation of splicing-mediated factors to precursor mRNA. This procedure is mediated by diverse regulatory processes including exon skipping, alternative 5′ or 3′ usage, mutually exclusive exons, intron retention, and use of alternative first or last exons. The production of cancer-specific transcripts created by alternative splicing is implicated in cancer biological processes; apoptosis, cell cycle, metabolism, invasion, proliferation, and angiogenesis. Light yellow, light blue and light orange rectangles indicated basal exons. Yellow rectangle indicated newly integrated exon. Pre-mRNA—precursor mRNA; SS—splicing site; SR protein—Serine/arginine—rich protein; ESE—exonic splicing enhancers; ESS—exonic splicing silencers; ISE—intronic splicing enhancers; ISS—intronic splicing silencers; hn-RNP—heterogeneous nuclear ribonucleoproteins.

Previous research has found that 60% of alternatively spliced variants encode functionally distinct proteins [29,30]. In a typical splicing process, the removal of the intron region is mediated by major and minor spliceosomes composed of numerous splicing factors containing five uridine-rich small nuclear RNAs (snRNA U1, U2, U4, U5, and U6) and functional analogs of the major spliceosome snRNAs (U11, U12, U4atac, and U6atac) [31]. The splicing process comprises consecutive reactions that involve the assembly of spliceosome components and their interaction with cis-acting regulatory sequences [32]. To begin, snRNP U1, SF1, and U2AF bind to the intron 5′ end splicing donor site (GU), intronic branch point, and intron 3′ end splicing acceptor site (AG), respectively. Then, U2 binds to the branch point site instead of SF1, and U4/U5/U6 snRNPs form networks with U1 and U2. After the release of U1 and U4, the activated spliceosome stimulates the cleavage of the intron 5′ and 3′ ends by forming a lariat, the release of the lariat/U2/U5/U6 complex, and the joining of post-spliced exons [6,31]. However, cancers tend to choose a substitutive pathway, cancer-specific alternative splicing, that generates several mRNA transcripts from the same gene locus with potentially different genetic functions, known as isoforms [33]. The differentially expressed isoforms in cancer, known as cancer-specific transcripts, are caused by alterations in internal or external factors. Subsequently, various biological processes that promote cancer development have changed (Figure 1) [34].

### 2.1. Cancer Promoting Mutations in Alternative Splicing Causing Factors

Fine-tuning between cis-acting elements and trans-acting factors coordinates alternative splicing networks [35]. Cancers are significantly affected by abnormal splicing events caused by somatic mutations in cis-acting splicing sequences and irregular alteration of trans-acting elements, including different activities of regulatory splicing factors and mutations in the core components, for example, RNA-binding proteins (RBPs), of the splicing machinery [36]. Changes in core splicing factors are related to cancer pathologies through dysregulation of cancer signaling transduction [37]. Numerous studies have identified that mutations in SRSF1, SRSF2, SF3B1, and U2AF1, which are essential components of the major spliceosome, are associated with the development of several types of cancers. SRSF1, which is upregulated in breast cancer, induces SRSF1-regulated alternative splicing events, such as exon inclusion and skipping, by binding to the 5′ or 3′ splice site. Overexpression of the exon-9-included CASC4 variant by SRSF1-regulated alternative splicing, which is considered a potential target for therapeutic development, increased proliferation, and decreased apoptosis [38]. Another example is the mutation of the splicing factor SRSF2, especially correlated with blood cancer types, which accelerates differential splicing of hn-RNP proteins in the SRSF2P95H mutant cell line [39,40] or alteration of RNA binding affinities [41]. Cancer-associated mutations in SF3B1 induce aberrant splicing of specific genes, such as *DVL2*, a regulator of Notch signaling, or disrupt interactions with other collaborating proteins, DDX42 and DDX46 [42,43]. In addition, mutation of U2AF1 in the S34 mutation hotspot region adjusted the progress of noncanonical translation by causing alternative splicing [44].

Furthermore, other types of RBPs contribute to dysregulated alternative splicing in cancer. For example, RNA-binding motif proteins adjust alternative splicing in cancer cells. Breast cancer-specific expression of SRPK1 accumulates phosphorylated RBM4 in the cytoplasm and then increases RBM4-regulated splicing transcripts of IR-B and MCL-1S [45]. RBM5, which is downregulated in bladder cancer, inhibits apoptosis [46], and RBM10 suppresses endometrial cancer proliferation by causing VEGFA alternative splicing [47]. Likewise, RBM6 represses the growth and progression of tumors and laryngocarcinoma by decreasing the expression of EGFR, extracellular signal-regulated kinase (ERK), and phosphorylated (*p*)-ERK [48]. Taken together, mutations accumulated in alternative splicing-causing factors lead to functional loss or change in the basal splicing process, thus contributing to the generation of cancer-specific splicing variants.

### 2.2. Cancer-Specific Transcripts Generated from Several Mechanisms of Alternative Splicing

Alternative splicing events affect the genetic flexibility and adaptability of cell biology in healthy cell metabolism, whereas in cancer, alterations in cancer cell processes occur via adjustment of apoptosis, invasion, proliferation, angiogenesis, and dysregulated metabolism. Through earlier computational analyses and microarray experiments, it has been revealed that alternatively spliced isoforms under oncogenic circumstances have a close relationship with cancer mechanisms involved in onset and development [49,50,51,52]. A list of cancer-specific transcripts that are abnormally expressed by alternative splicing and continuous regulatory processes is listed in Table 1. These previous findings have shown that the expression of cancer-specific transcripts, which encode major factors modulating important biological processes, is increased or decreased by alternative splicing mechanisms such as exon skipping, alternative 5′ or 3′ SS usage, mutually exclusive exons, intron retention, and usage of alternative first or last exons. The altered expression of abnormal transcripts promotes cancer progression by disrupting the normal regulatory system via multiple pathways (Figure 1).

## 3. Regulation of Cancer Related-Biological Processes by TE Induced Alternative Splicing

**Figure 2:**
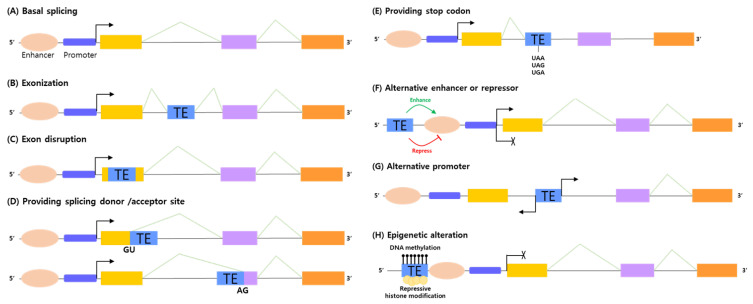
The induction mechanisms of alternative splicing by integrated TEs (**A**) basal splicing process without TE integration. Insertion of TE is interrelated with the progression of alternative splicing through induction mechanisms; (**B**) exonization, (**C**) exon disruption, (**D**) providing splicing donor/acceptor sites, (**E**) providing stop codons, (**F**) alternative enhancer or repressor, (**G**) alternative promoter, and (**H**) epigenetic alteration. Yellow, light purple and orange rectangles indicated basal exons.

Inserted TEs modulate numerous biological processes including development [95], adaptive evolution [96], and disease progression, including cancer [97]. After integration of the genome, some activated TEs can modify the expression or transcriptional responsiveness of specific genes by stimulating alternative splicing through certain processes, such as: exonization; exon disruption; providing splicing donor/acceptor sites; alternative regulatory regions; premature stop codons; and inducing epigenetic alterations at the transcriptional level (Figure 2). Previous studies have found that disease biological pathways are affected by disease-specific transcript variants triggered by TE insertion [98,99]. In particular, overexpression of TEs is closely correlated with the onset and development of cancer by causing abnormal alterations in cancer progression.

### 3.1. Exonization and Exon Disruption

In a process called exonization, TEs can integrate into genomic regions and offer recognition by the splicing machinery as a newly recruited exon [100]. Approximately 4% of human genes contain TE motifs in their coding regions, indicating that exons may have been derived from the exonization of TEs [101,102,103,104,105,106]. Some studies have identified that exonized LINEs in the human genome provide an additional domain and produce abnormal transcripts through diverse alternative splicing mechanisms in cancers. For example, overexpression of the cancer-specific cadherin-12 (CDH12) variant, a subtype of the N-cadherin family, can be generated by somatic LINE-1 insertion and induces migration and invasion of colon cancer cells by targeting the transcription factor, Snail [99,107]. Other researchers have also found that overexpressed LINE-1 *MYC* products are characterized in breast cancer patients [108,109]. Furthermore, Alu inserted into the coding region can act as an alternative exon in lung cancer-related genes. One study showed that the expression of the canonical *ADAR2* transcript, which functions as a tumor suppressor gene, was downregulated and other aberrant transcripts were inversely upregulated. The TE-derived isoforms among these aberrant transcripts are formed by the inclusion of an alternative exon 5a, which introduces a 120-nucleotide coding Alu-repeat sequence [110,111]. According to other studies, Alu can also integrate into *MYH11*, which is important for cell migration and adhesion by encoding smooth muscle myosin and make longer isoforms by adding additional exons. This insertion led to a frameshift mutation and over-production of a truncated protein [112,113].

In contrast, some proportion of inserted TEs into the coding sequence result in complete loss-of-function mutations and drastic changes in the encoded proteins by prompting gene disruption under oncogenic circumstances [114]. As a representative example, mutations in *BRCA2*, mainly observed in breast and ovarian cancers, could be induced by the insertion of Alu elements. The Alu element, integrated into the coding region of *BRCA2*, resulted in the elimination of the targeted exon 3 from the equivalent mRNA molecule by target site duplication, containing a specific 9 bp long segment as a recognition site for the transposition machinery [115]. Additionally, other studies have shown that TRPC6, which is important for cell proliferation and migration, is strongly expressed in breast cancer epithelial cells. The disrupted transcript produced by the LTR insertion is overexpressed in breast cancer [116,117]. In a systemic analysis, two genes, *SHSC1* and *KLK2*, were abnormally spliced through an internal exon skipping mechanism after LINE-1 integration [112]. In particular, some TEs activate gene disruption, which promotes oncogenic processes via integration with tumor suppressor genes. The tumor suppressor gene, *LRP1B*, which suppresses cancer cell growth and invasion, was downregulated in colon cancer patients and 19 retrotransposon insertions were observed [118]. The data show that these genetic alterations are caused by exonization or genetic disruption, which adjusts the proportion of variants in a biological direction favorable to the occurrence and growth of cancer.

### 3.2. Providing Splicing Donor/Acceptor Site and Stop Codon

TEs are often composed of many splice-donor and acceptor sites, which lead to irregular splicing processes by interacting with splicing factors and RBPs. RBPs serve site preferences, which prompt them to reach specific regions of TEs [119,120]. TEs change the expression of cancer-related gene variants through the suggestion of alternative 5′ or 3′ SSs. In particular, inserted Alu retroelements, which contain multiple sites with sequences similar to those of SSs, could be considered a real exon by offering pseudo-SSs [101,102,121,122]. One study identified that PDZK1, which plays a crucial role in ion-channel organization, upregulates gene expression by providing alternative 5′ sites via the inserted Alu [123,124]. Other studies have also found that Alu offers 5′ alternative sites to the tumorigenic gene, *HINFP*, which activates cyclin E/CDK2 in the cell cycle and regulates DNA damage-induced cell cycle checkpoints [124,125,126].

The insertion of TE inside the intronic and coding regions of a premature mRNA can introduce an irregular stop codon or polyadenylation signal, resulting in truncated transcripts. Human antigen R (HuR) or fused in sarcoma (FUS) proteins, characterized by binding preference to U-rich motifs, alternatively bind to inserted TE regions and induce the nonsense-mediated decay process [127]. One research team confirmed that a short variant isoform of *CHM* was generated from the insertion of LTR12C as a carrier of an early stop codon and was highly expressed in colon cancer cell lines and tumor samples [128]. Furthermore, LINE-1 elements retain a polyadenylation signal within their own sequences, and AATAAA sequences are usually generated in the A-rich tail region of SINEs and LINEs [129]. The LINE-1 insertion into the last exon of *APC*, a tumor suppressor gene, led to disruption through the proposal of a polyadenylation site and was associated with the development of sporadic colorectal tumors [130]. Another study has also shown that germ line L1 insertions into *MCC* as an upstream inhibitor of the Wnt/β-catenin pathway can repress the expression of MCC and overexpress the β-catenin protein. This study suggests a functional link between L1 insertions and HCC-predisposing mutations [131]. Specific variants created by alternative SSs and stop codons are not only used as potential cancer diagnostic biomarkers but also for therapeutic applications.

### 3.3. Providing Alternative Regulatory Sequences Such as Enhancer, Repressor and Promoter

TEs, especially major families of retrotransposons, including LINE and SINE, are involved in the adjustment of upstream open reading frame-related genetic expression by operating cis-acting elements, such as promoters, enhancers, and repressors, to control gene expression [132,133]. TE insertion can boost the upregulation of the cis-open reading frame, causing the stimulation of oncogenic traits for cancer development. For example, MAD1L1, a cell cycle regulator, has an LTR sequence-derived promoter as one of two promoters. Isoforms induced by the LTR promoter were abundant in various tumors compared to the universally expressed form and increased cancer cell proliferation [134]. Similarly, carbonic anhydrase, which is relevant to ion, fluid, and acid-base balance, CA1, is over-expressed in colon cancer by the LTR-derived primary promoter [134,135]. In addition, an alternative promoter generated by LINE-1 insertion elevates the expression of DBC-1, which acts as an interface between apoptosis and colon cancer progression by controlling wnt/β-catenin-mediated expression of *MACC1* [99,136]. Additionally, the LINE-1 integrated region into SYT1 acts as an alternative promoter and upregulates SYT1 expression, which over-activates the regulatory mechanism in membrane interactions in lung cancer [137].

On the contrary, TEs can contribute to cancer development by promoting expression of the truncated isoform after insertion, thereby downregulating the original function of the corresponding gene. For example, ARID3A has a tumor suppressive function that inhibits somatic cell reprogramming according to loss-of-function analysis [138]. According to previous research, the Alu sequence inserted into the regulatory region of *ARID3A* sequences increased the production of truncated proteins, which are unable to repress dedifferentiation in lung cancer cells [139]. Furthermore, some studies have indicated that TE-derived promoters activate tyrosine kinase receptors as oncogenes in colon cancer. The proto-oncogene *MET1* is controlled by an alternative LINE-1 promoter within the canonical intron 2 by increasing the abnormal isoform translated into a truncated protein [140,141]. In another case, the LTR-derived alternative promoter accelerated ERBB4 expression, which alters cell proliferation and differentiation, migration, and apoptosis, resulting in an increase in the truncated isoform [99,142]. TE-derived regulatory regions have changed the overall expression level of transcripts by upregulating or downregulating cis-acting genes and are involved in diverse cancer-specific biological changes.

### 3.4. Epigenetic Alteration

TEs, mostly silenced in normal situations, lose their repressed markers, such as DNA methylation and suppressive histone modifications, by epigenetic dysregulation in cancer cells [143]. Many tumors have over-expressed the DNA demethylating enzymes TET2 and TET3, which result from higher ERV expression. That is, the demethylation of specific genes might be directly related to the transcription level of TEs, including ERV [144]. In renal cell cancer, DNA hypomethylation also activated TEs, ERVs expression, and immune signaling [145]. Another study also discovered that hypomethylation of retrotransposons can lead to their activation and translocation to other regions of the genome and stimulate an increase in genomic instability in T-cell lymphoma [146]. Likewise, several studies have focused on the activation of LINE-1 elements by demethylation of their own sequences linked to the development of cancers [147,148,149], such as prostate carcinoma and hepatocellular carcinoma [150,151,152]. In accordance with transcriptome analysis for chronic lymphocytic leukemia, the results suggest that TEs are globally hypomethylated compared to normal tissues [153]. In melanoma, LINE-1 hypomethylation was also closely correlated with the shortened period of relapse and survival time of patients and was connected with the metastatic conversion of primary cancer [154].

Additionally, when TEs are inserted into a tumor suppressor gene, they may cause cancer by providing new methylation or histone modifications to regulatory sequences [143]. Numerous studies have shown that inserted TEs can spread repressive epigenetic markers to regions adjacent to genomic sequences. Based on previous studies, TEs, especially highly repetitive Alu elements, are regarded as methylation centers in the genome [155,156]. Through epigenetic pattern analysis, one study revealed that Alu, integrated into intron 1, might offer additional methylation to *MLH1* in correlation with trans-acting elements. Expression of MLH1, which is closely associated with mismatch repair, might be downregulated by hypermethylated Alu elements and is predicted to be closely associated with cancer development [157]. As carriers of epigenetic markers, TEs can integrate into the genomic region and generate tumorigenic status by changing the location and proportion of epigenetic markers in the regulatory region.

## 4. MDTEs as Regulatory Elements Linking Alternative Splicing by TE Integration and Cancer Organically

### 4.1. MDTEs’ Regulatory Processes Related to Cancer Progression and Cancer Therapy

**Figure 3:**
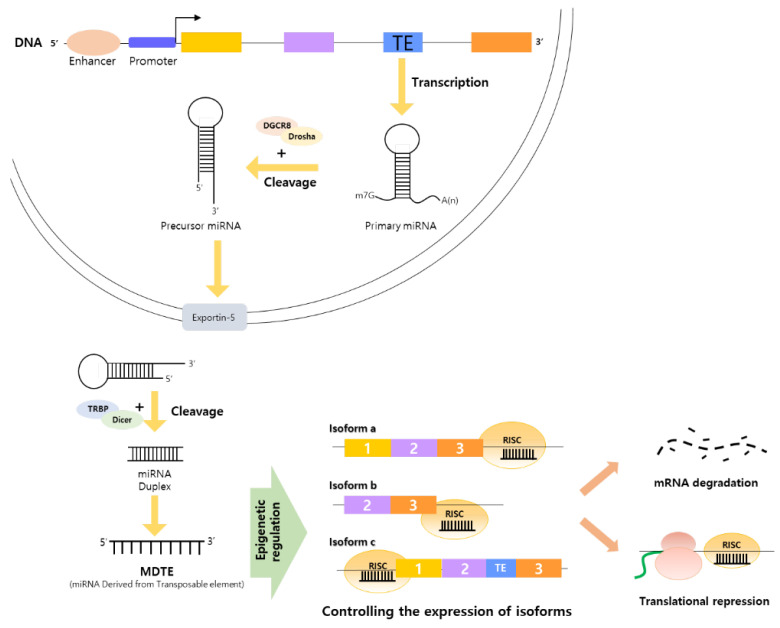
General regulatory process of miRNAs derived from TEs to control the expression of oncogenic transcripts at the post-transcriptional level. RISC-RNA-induced silencing complex. Yellow, light purple and orange rectangles indicated basal exons.

Inserted TEs occasionally produce miRNAs from their sequences [24]. The TE sequence is transcribed as a primary miRNA and processed into precursor miRNAs through cleavage by proteins such as DGCR and Drosha. Precursor miRNAs move to the cytoplasm through the nuclear transport protein exportin-5 and are cleaved by proteins including TRBP and Dicer to form a miRNA duplex. One of the two complementary strands creates an RNA-induced silencing complex with functional proteins and binds to mRNA for mRNA degradation or translational repression. Through epigenetic regulation, MDTEs act as regulators that control the expression of several alternative transcripts (Figure 3) [158,159]. The MDTEs adjust the portion of oncogenic transcripts not only through basic inhibitory mechanisms but also through interactive mechanisms by interacting with other genetic elements, such as long non-coding RNA, transcription factors, and circular RNA [160,161,162,163]. Overexpression or knockdown of genes required for oncogenic processes under the adjustment of MDTEs had an effect on cancer onset and progression [164,165]. In brief, TE insertion affects pre-transcriptional regulation by directly participating in the alternative splicing process and has a big impact on fine-tuning the expression of transcripts at the post-translational level by creating a regulatory element, called miRNA, from their sequences.

Numerous studies have indicated that miRNAs are closely related to the expression of cancer-related genes as significant regulatory factors through the repression or activation of their target genes, contributing to the onset and development of human cancer types [166,167,168]. Oncogenic miRNAs in previous studies have confirmed that a substantial proportion of miRNAs known to affect the development of cancer are derived from TEs. Despite the importance of biological correlations, few studies on the interactive relationship between MDTE and cancer progression have been conducted.

According to previous research, TEs are important drug targets in the field of disease treatment because they extend from disease-causing factors [169,170,171]. Particularly in cancer, TEs have been applied as major drug targets for cancer treatment for the design of anticancer drugs. In prostate cancer, reverse transcriptase (RT), the enzyme which is encoded as region of the open reading frame 2 of LINE-1, is used as a target for anti-prostate cancer drugs. Inhibitors of reverse transcriptase (RT) repressed the proliferation of cancer cells and tumor progression and inversely promoted differentiation in animal models [172,173]. TEs were also used as vital coordinators related to cancer therapy by generating miRNAs from their sequences. Exosomal MDTEs can modulate cancer cell resistance, leading to tumor recurrence by regulating the chemosensitivity of cancer cells, which are promoted by altered cellular signaling pathways in chemotherapy [174]. As a representative example, exosomal miR-151a produced by LINE controls drug resistance in glioblastoma and pancreatic adenocarcinoma. Exosomal miR-151a sensitized temozolomide (TMZ)-resistant glioblastoma cells to TMZ by interacting with X-ray repair cross-complementing 4 (XRCC4) [175]. In pancreatic ductal adenocarcinoma, macrophage-derived exosomal miR-151a decreases the sensitivity of cancer cells to gemcitabine, dramatically [176]. Although there are still several technical limitations in the clinical trial of cancer using TEs and MDTEs, they are crucially considered as a future drug target.

### 4.2. Cancer Controlling MDTEs in the Top 5 Mortality Cancers

According to the global cancer report of 2020, lung cancer is the leading cause of cancer death (18% of the total cancer-related deaths), followed by colon (9.4%), liver (8.3%), stomach (7.7%), and breast (6.9%) cancers for both sexes combined [177]. Table 2 indicates the MDTEs dysregulated in five major mortality-causing cancers in the previously published literature over the last five years (2018–2022). The information on the origin of the specific TE sequence of each miRNA was organized with regard to the information of the human genome 38 registered in the UCSC genome browser. MDTEs can change cancer pathways by regulating the expression of their direct target genes on the basis of interactions with various genetic factors, leading to cancer induction or progression.

#### 4.2.1. Lung Cancer

Lung cancer is the second most frequently diagnosed cancer with the highest death rate in 2020 [177]. MDTEs have been considered more sensitive potential prognostic biomarkers and therapeutic indicators in lung cancer than other tumor markers. Overexpressed hsa-miR-421 and hsa-miR-1290, both derived from LINE (L1) and DNA transposons, respectively, were linked to a poor prognosis and development of lung cancer, including advanced tumor stage, enlarged tumor size, lymph node involvement, and distant metastasis [178,179]. Increased or decreased miRNAs can alter the biological mechanisms of lung cancer into a favorable environment for cancer development by modulating the expression of their target genes. For instance, upregulated miR-4317 functions as a potential suppressor of lung cancer by directly binding fibroblast growth factor 9 and cyclin D2, resulting in the inhibition of proliferation, colony formation, migration, and invasion [180]. Similarly, miR-1246 prevented cell invasion and epithelial mesenchymal transition by interacting with C-X-C chemokine receptor type 4 and blocking the JAK/STAT and PI3K/AKT signal pathways in lung cancer cells [181].

In-depth studies have shown that MDTEs are involved in substantive cancer treatment. For example, miR-181b and miR-885-3p are closely related to chemoresistance by targeting BCL2 and Aurora A, respectively [182,183]. In some cases, miRNA regulation in lung cancer has a molecular connection with non-coding RNA or circular RNA. According to one study, MALAT1, an upregulated long non-coding RNA, promotes proliferation, apoptosis, migration, and invasion in non-small cell lung cancer by downregulating miR-374b-5p and inversely upregulating SRSF7 [184]. Another study also revealed a correlative axis among circular RNA, target mRNA, and miRNAs. Additionally, Circ-ZKSCAN1 increased FAM83A expression and restrained MAPK signaling by targeting carcinogenic miR-330-5p as a sponge to aid non-small cell lung cancer progression [185].

#### 4.2.2. Colon Cancer

Colon cancer is a secondary cause of death and the third most frequently diagnosed- cancer type worldwide in both sexes [177]. Biological processes regulated by miRNAs are connected with colon cancer incidence and development. Cell growth and proliferation were stimulated by downregulation of miR-583-3p and miR-1273g-3p, inducing overexpression of their target genes *PSME3* and *MAGEA3/6* in colon cancer cells [186,187]. Some studies have found that exosome-transmitted miRNA-335-5p and miR-340-5p, directly controlled by LncRNA LINC00662, promote colorectal cancer invasion and metastasis through facilitating epithelial-mesenchymal transition and activating the ERK signaling pathway [188,189]. Moreover, angiogenesis in colon cancer has been over-activated by miR-181a, broadly known as an oncogenic miRNA that stimulates over-activation of VEGF signaling in various cancer types [165,190]. In addition, miR-552 serves as an indicator of poor prognosis in cancer patients and is a potential diagnostic target by regulating the expression of PTEN [191].

Some miRNAs derived from LINE act as broad tumor suppressor miRNAs related to tumor hallmarks such as proliferation, growth, apoptosis, and migration. Downregulated expression of miR-708 in various cancers, including colorectal cancer tissues and cell lines, activates proliferation and metastasis and inhibits apoptosis via the targeting of ZEB1 through the Akt/mTOR signaling pathway [192]. Additionally, miR-28-5p was identified as a component of the combined regulatory axis UCA1/miR-28-5p/HOXB3, which controls tumor size and stage, cell growth, and migration in colon cancer [193].

#### 4.2.3. Liver Cancer

Liver cancer is the third leading cause of cancer-associated deaths worldwide. Liver cancer has a poor prognosis for late diagnosis at advanced and metastatic stages without representative prior symptoms and sufficient therapeutic approaches [194]. Studies of diagnostic biomarkers are important for the early diagnosis of liver cancer. One study showed that upregulated exosomal miR-224 derived from the DNA transposon is a diagnostic and prognostic biomarker of hepatocellular carcinoma, resulting in a lower survival rate and increased proliferation and invasion [195]. Moreover, overexpression of miR-493-5p and miR-608 suppresses the proliferation and invasion of liver cancer cells by regulating the expression of their target genes, *VAMP2* and the BET family protein BRD4, respectively [196,197]. Some miRNAs originating from SINE negatively control the Warburg effect, a distinctive metabolic phenomenon that favorably utilizes glucose through aerobic glycolysis by silencing their target genes [198]. miR-342-3p, a tumor suppressor miRNA, inhibits cancer cell proliferation by inactivating the IGF-1R-mediated PI3K/AKT/GLUT1 signaling pathway. Suppression of IGF-1R weakens glycolysis by decreasing glucose uptake, lactate generation, ATP production, and extracellular acidification rate, inversely increasing the oxygen consumption rate in hepatoma cells, causing activation of proliferation [199]. Another study demonstrated that forced expression of miR-885-5p enhanced aerobic glycolysis by reducing glucose uptake and lactate production through inhibition of hexokinase 2, which catalyzes the first step of glycolysis [200].

Liver cancer-controlling miRNAs also have a close correlation with long non-coding RNAs and form a regulatory axis with other factors, including transcription factors and target genes of miRNA, based on the competing endogenous RNA hypothesis that lncRNAs might act as a molecular sponge for miRNA. One study has identified that lncRNA H19 and miR-326 are expressed inversely in hepatocellular carcinoma and control the expression of TWIST1, a downstream target of miR-326, tempting changes in cancer cell growth, migration, and invasion [201]. Another study confirmed a negative correlation between lncRNA MIR31HG and miR-575. MIR31HG suppresses proliferation and invasion of liver cancer cells by inhibiting miR-575, an upstream regulator of the tumorigenicity 7-like (ST7L) gene [202].

#### 4.2.4. Stomach Cancer

Stomach cancer, the most common malignant tumor, ranks fifth in incidence and fourth in mortality in global cancer statistics for 2020 [177]. The onset of stomach cancer is often caused by the abnormal expression of specific genes. Several studies have confirmed that changes in the expression of cancer-related genes can be regulated by MDTEs. Consequently, MDTEs can control cancer biological pathways and manage cancer progression. Both miR-585 and miR-1269, are derived from the LTR element and inversely regulate the proliferation of stomach cancer cells. The tumor suppressor miRNA miR-585 binds to MAPK1 and prevents its expression, leading to suppression of tumor proliferation and migration [203]. On the other hand, oncogenic miRNA miR-1269 promotes proliferation and cell cycle G1-S transition by activating the AKT signaling pathway while suppressing apoptosis by targeting RASSF9 via the Bax/Bcl-2 signaling pathway [204]. Similarly, upregulated LINE derived-miR-552 also functions as an oncogenic miRNA, as an accelerator of gastric cancer progression, increased metastasis, and worsens therapeutic outcomes by targeting forkhead box O1 (FOXO1) and modulating the PI3K/AKT pathway [205]. Cancer cells induce polymorphisms in major oncogenes to circumvent this regulatory mechanism of MDTE. A recent study found that MUC4, a regulator of cell apoptosis and tumorigenesis, is aberrantly expressed in numerous cancer types. The evaluated expression of the rs2641726 C allele of MUC4 was significantly concerned with cancer incidence by providing a binding site to attenuate its interaction with miR-581 [206].

In addition, long non-coding RNAs and circular RNAs also contribute to the correlation between MDTEs and their target genes in gastric cancer. One study has verified that the novel abundantly expressed lncRNA RP11-290F20.3, named GC-related lncRNA1 (GCRL1), could change gastric cell proliferation and metastasis both in vitro and in vivo by sponging the tumor suppressor miRNA miRNA-885-3p and stimulating overexpression of the target gene cyclin-dependent kinase 4 (CDK4) [207]. In addition, long non-coding RNAs such as TRPM2-AS and LINC00324 act as miRNA sponges for MDTEs such as miR-612 and miR-3200-5p and attenuate tumorigenesis by increasing the expression of their target genes [208,209]. Furthermore, circular RNAs, Circ_0008287, and Circ-LDLRAD3, boost immune escape mechanisms or cancer cell viability criteria such as cell growth, migration, and invasion by regulating MDTEs, miR-548c-3p, and miR-224-5p, its target genes axis in stomach cancer [210,211].

#### 4.2.5. Breast Cancer

Breast cancer is the most common cancer diagnosed in women and the most prevalent cancer in 2020 in both sexes [177]. Improved survival outcomes of breast cancer are associated with understanding the molecular processes driving breast cancer development, including the interaction of miRNA and target mRNA. For example, miR-421 is a valuable diagnostic biomarker that adjusts breast cell proliferation through targeting RDCD4 [212,213]. According to other studies, as a tumor suppressor miRNA, miR-326, and miR-340-5p derived from DNA transposon controlled vital tumor pathways, ErbB/PI3K and Wnt/beta-catenin signaling pathways [214,215].

In particular, a few miRNAs facilitate tumorigenesis of triple-negative breast cancer, which is a subset categorized by the negative expression of human epidermal growth factor receptor 2, estrogen, and progesterone receptors, and is also considered one of the highest-risk and poorest prognostic subtypes of breast cancer [216,217]. LINE-originated miRNAs, miR-582-5p, and miR374-5p, stimulate cancer invasion and metastasis by antagonizing their target genes, *CMTM8* and *ARRB1* [218,219]. Moreover, upexpressed miR-224-5p, derived from the DNA transposon in triple-negative breast cancer cells, enhances cell proliferation, migration, and invasion by inhibiting CASP9 [220].

**Table 2:** The list of miRNAs dysregulated in five major mortality-causing cancers in the last 5 years.

Cancer Type	MiRNA	Subclass	Superfamily	Target Gene	Reference
**Lung cancer**	hsa-miR-421	LINE	L2	-	[178]
	hsa-miR-4317	SINE	MIR	*FGF9*, *CCND2*	[180]
	hsa-miR-330-5p	SINE	MIR	*RASSF1A*	[221]
				*FAM83A*	[185]
	hsa-miR-374b-5p	LINE	L2	*SRSF7*	[184]
	hsa-miR-544a	DNA transposon	hAT-Charlie	*FBXW7*	[222]
	hsa-miR-1183	LINE	L2	*PDPK1*	[223]
	hsa-miR-181a	LINE	RTE-BovB	*GAS7*	[224]
	hsa-miR-181b	LINE	RTE-BovB	*Bcl-2*	[182]
	hsa-miR-340	DNA transposon	TcMar-Mariner	-	[225]
	hsa-miR-340-5p	DNA transposon	TcMar-Mariner	*KPNA4*	[226]
	hsa-miR-885-3p	SINE	MIR	*Aurora A*	[183]
	hsa-miR-378a-3p	SINE	MIR	*CDK4/CDK6*	[227]
	hsa-miR-1246	LTR	ERVL-MaLR	*CXCR4*	[181]
	hsa-miR-1290	DNA transposon	TcMar-Tigger	-	[179]
	hsa-miR-326	DNA transposon	hAT-Tip100	hsa_circ_0003998	[228]
				*Sp1*	[229]
	hsa-miR-608	LINE	L2	*MIF*	[230]
**Colon cancer**	hsa-miR-585-3p	LTR	ERVL-MaLR	*PSME3*	[186]
	hsa-miR-335-5p	SINE	MIR	*RASA1*	[188]
	hsa-miR-181a	LINE	RTE-BovB	*SRCIN1*	[190]
	hsa-miR-708	LINE	L2	*ZEB1*	[192]
	hsa-miR-1273	SINE	Alu	*MAGEA3*/6	[187]
				*circPIP5K1A*	[231]
	hsa-miR-340-5p	DNA transposon	TcMar-Mariner	*CLDN8*, *IL22*	[189]
	hsa-miR-552	LINE	L1	*PTEN*, *p53*	[191]
	hsa-miR-28-5p	LINE	L2	*HOXB3*	[193]
	hsa-miR-374b-5p	LINE	L2	*LRH*-*1*	[232]
**Liver cancer**	hsa-miR-23c	SINE	MIR	*ERBB2IP*	[233]
	hsa-miR-575	SINE	MIR	*ST7L*	[202]
	hsa-miR-608	LINE	L2	*BRD4*	[197]
	hsa-miR-326	DNA transposon	hAT-Tip100	*TWIST1*	[201]
	hsa-miR-645	DNA transposon	hAT-Charlie	*SOX30*	[234]
	hsa-miR-493-5p	LINE	L2	*VAMP2*	[196]
	hsa-miR-224	DNA transposon	DNA transposon	*GNMT*	[195]
	hsa-miR-342	SINE	tRNA-RTE	*IGF*-*1R*	[199]
				*MCT1*	[235]
	hsa-miR-378a	SINE	MIR	*VEGFR*, *PDGFRβ*, *c*-*Raf*	[236]
	hsa-miR-885-5p	SINE	MIR	*HK2*	[200]
	hsa-miR-421	LINE	L2	*MAPK14*	[237]
**Stomach cancer**	hsa-miR-575	SINE	MIR	*PTEN*	[238]
	hsa-miR-581	DNA transposon	hAT-Charlie	*MUC4*	[206]
	hsa-miR-552	LINE	L1	*FOXO1*	[205]
	hsa-miR-885-3p	SINE	MIR	*CDK4*	[207]
	hsa-miR-421	LINE	L2	Hsacirc0001546	[212,239]
	hsa-miR-181a	LINE	RTE-BovB	caprin-1	[240]
	hsa-miR-612	SINE	MIR	*IGF2BP1*, *FOXM1*	[208]
	hsa-miR-224-5p	DNA transposon	DNA transposon	*circ-LDLRAD3*	[211]
	hsa-miR-3200-5p	LTR	ERVL	*BCAT1*	[209]
	hsa-miR-1269	LTR	ERVL	*RASSF9*	[204]
	hsa-miR-585	LTR	ERVL-MaLR	*MAPK1*	[203]
	hsa-miR-4317	SINE	MIR	*ZNF322*	[241]
	hsa-miR-548c-3p	DNA transposon	TcMar-Mariner	*CLIC1*	[210]
**Breast cancer**	hsa-miR-130a-3p	LINE	RTE-BovB	*FOSL1*, *RAB5B*	[242]
	hsa-miR-582-5p	LINE	CR1	*CMTM8*	[218]
	hsa-miR-224-5p	DNA transposon	DNA transposon	*CASP9*	[220]
				*Smad4*	[243]
	hsa-miR-1246	LTR	ERVL-MaLR	-	[244]
	hsa-miR-326	DNA transposon	hAT-Tip100	*EGFR, ErbB2, ErbB3, AKT1, AKT2, AKT3*	[214]
	hsa-miR-708	LINE	L2	-	[245]
	hsa-miR-374a-5p	LINE	L2	*ARRB1*	[219]
	hsa-miR-335-5p	SINE	MIR	EphA4	[246]
	hsa-miR-181a	LINE	RTE-BovB	*AK024094*	[161]
	hsa-miR-340-5p	DNA transposon	TcMar-Mariner	*LGR5*	[215]
	hsa-miR-421	LINE	L2	*PDCD4*	[213]

### 4.3. Cancer Regulatory MDTEs in Other Cancer Types

In addition, it has been confirmed that MDTEs can function as vital controlling factors in other cancer types (Table 3). In renal cancer, DNA transposon-derived hsa-miR-224 in collaboration with miR-193a-3p promotes cell proliferation and migration by targeting al-pha-2,3-sialyltransferase IV and activating the PI3K/AKT pathway [247]. Similarly, miR-340 prevents the proliferation, migration, and invasion of squamous cell carcinoma cells by directly targeting the Ras homolog gene family member A [248]. In bladder cancer, miR-374 and miR-612 function as tumor suppressor miRNAs through interaction with *ZEB2* and malic enzyme 1 [249,250]. Upregulated miR-582-5p directly targets AKT3 and affects cell proliferation and apoptosis in endometrial carcinoma [251]. LINE-originated miRNAs, miR-1271, 887-3p, and miR-552, regulate the progression of pancreatic and ovarian cancers by inhibiting each target gene [252,253,254]. Some MDTEs have the same effect on cancer as their regulators. In cancer studies, miR-224 and miR-374b were shown to be oncogenic miRNAs and tumor suppressor miRNAs, respectively, in cervical cancer by binding to pentraxin 3 and junctional adhesion molecule-2, respectively [255,256].

In addition, MDTEs have a significant effect on chemotherapy as well as their role as regulators of cancer progression. For example, the expression of the circulating miRNA, miR-1290, was downregulated in the plasma of oral squamous cell carcinoma patients compared to that in healthy volunteers. According to clinicopathological and Cox regression analyses, oral cancer patients with lower expression of miR-1290 showed poor pathological response to preoperative chemoradiotherapy and a lower five year overall survival rate. As a valuable biomarker, circulating miR-1290 can predict the clinical response to chemoradiotherapy and the overall survival rate in patients with oral squamous cell carcinoma [257]. Poor survival rates by increasing chemoresistance were caused by LTR-derived miR-1246, overexpressed in oral cancer patient tissues. Moreover, miR-1246 represses CCNG2 expression, leading to cancer cell stemness progression, which can represent relapse and metastasis [258]. Thus, MDTEs can be applied as therapeutic and diagnostic biomarkers, as well as expression regulators of oncogenes promoting cancer development and tumor suppressor genes inhibiting the generation of tumors. However, their biological and clinical value in patients with cancer has not yet been fully explored, and few research papers illuminating the relationship between MDTE and cancer have been conducted.

## 5. Conclusions

**Figure 4:**
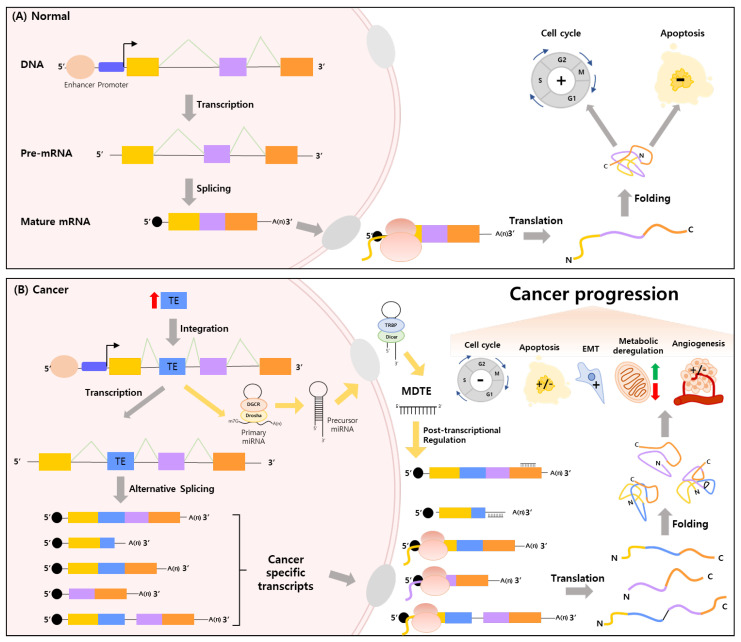
Impact of cancer-specific alternative splicing promoted by TE integration. Integrated TE is a critical regulator of many aberrant cancer-related biological processes implicated in cancer pathogenesis. (**A**) Fundamental splicing process in normal cell biology. (**B**) Macroscopic regulatory mechanism of cancer-specific transcript expression under the cancer environment. EMT: Epithelial-Mesenchymal Transition. Yellow, light purple and orange rectangles indicated basal exons.

This review focuses on the biological consequences of TEs as a key source of alternative splicing and as a vital transcriptional regulator of various oncogenic processes associated with the onset and development of cancer. Under normal conditions, RNA processing fidelity is preserved by basal splicing, which maintains normal physiological homeostasis. Conversely, with the incidence of cancer, disruption of regulatory homeostasis by TE insertion can produce cancer-specific transcripts and contribute to the development of cancer by altering the expression of cancer progression-related genes. Moreover, miRNAs derived from TE can regulate a portion of cancer-specific transcripts at the post-transcriptional level. That is, integration of TE acts as a critical regulator of many aberrant tumorigenic processes implicated in cancer pathogenesis, including the cell cycle, apoptosis, EMT, metabolic deregulation, and angiogenesis (Figure 4). Despite this scientifically revealed organic relationship, there have been few studies on the correlation between alternative splicing by inserted TEs and cancer-related MDTEs by analyzing in vivo data originating from samples of cancer patients. Hence, in-depth research and systematic analyses of these interactions are necessary to provide therapeutic insights into cancer treatment and a better understanding of oncogenic regulatory mechanisms from a macroscopic point of view.

## Figures and Tables

**Table 1:** Differential expression of cancer-specific isoforms produced by alternative splicing events that adjust cancer-related biological processes.

Cancer Related Biological Process	Type of Alternative Splicing	Gene	Protein	Isoform	Regulatory Process	Ref
**Apoptosis**	Alternative 5′ SS usage	BCL2L1	Bcl-2-Like Protein 1	**BCl-XL**	Through the alternative use of two competing 5′ SSs in exon 2, produced BCL-XL which has an antiapoptotic effect and functions as a dominant regulator.	[53,54,55]
	Exon skipping	SYK	Spleen Associated Tyrosine Kinase	**SYK(S)**	Switching SYK(L) to SYK(S) generated by exon 9 skipping induces apoptosis in ovarian cancer.	[56]
	Intron retention	STAT2	Signal Transducer and Activator of Transcription 2	**STAT2 + I19**	STAT2 + I19, splice variant containing intron 19 which has a stop codon before the Src homology 2 domain, leads to disruption of STAT dimerization and suppresses IFN-induced apoptosis in IFN-resistant cells.	[57]
	Exon skipping	ASPP2	Apoptosis-Stimulating of P53 Protein 2	**ASPP2K**	ASPP2K, which has a truncated C-terminal domain losing the p53 binding regions by exon skipping, possesses dominant-negative activity, impairing the induction of p53 dependent apoptosis and promoting cancer aggressiveness.	[58]
	Exon skipping	FAS	Fas Cell Surface Death Receptor	**sFAS**	An alternatively spliced isoform, soluble Fas (sFAS), generated by the skipping of exon 6 that encodes the transmembrane domain, cannot localize to the plasma membrane. As a result, upregulated sFAS inhibits the extrinsic pathway of apoptosis in various cancer types.	[59,60,61]
	Exon inclusion	MCL1	Myeloid Leukemia Cell Differentiation Protein Mcl-1	**MCL1-L**	Melanocytes upregulate MCL-1L, a splicing variant of MCL1 by exon 2 inclusion, in response to UVB radiation to protect themselves against apoptosis, whereas melanoma cells elevating MCL1-L expression without UV exposure are resistant to apoptosis.	[62]
**Invasion** **(EMT)**	Exon skipping	ENAH (MENA)	ENAH Actin Regulator	**MENA v6**	ENAH (known as Mena), controls actin nucleation as well as cell morphology and motility. Expression of the exon skipped splicing isoform, Mena11a, has been correlated with epithelial markers and decreased invasion. Inversely, increased expression of MenaINV by exon inclusion has been associated with mesenchymal markers and increased invasion and metastasis.	[63,64,65,66]
	Exon inclusion			**MENA INV**		
	Alternative 5′ SS usage	KLF6	Kruppel Like Factor 6	**KLF6-SV1**	KLF6-SV1 uses an alternative 5′SS, causing frameshift, and produces a protein isoform that contains 21 novel amino acids but lacking all three of the zinc finger domains. Upregulated expression of KLF6-SV1 increases cell survival, migration, and invasion in various cancer cells.	[67,68]
	Exon inclusion	CD44	CD44 Antigen	**CD44v8-10**	Expression of CD44v8-10, an alternative isoform including the variable portion of exon 8 to 10, induces a higher metastatic potential of cancer cells than the standard form of CD44 in breast cancer cell lines.	[69]
				**CD44v6**	CD44 variant including variable exon 6 (CD44v6) has been identified that promotes the development of metastasis by involving epithelial-mesenchymal transition in cancers.	[70,71]
	Alternative 5′ SS usage	CTNND1	p120-catenin	**p120-1A**	p120-catenin (p120ctn) isoforms produced by alternative 5′SS usage, p120-1A, and -3A, induced the EMT of tumor cells. Especially, in non-small cell lung cancer (NSCLC), both p120-1A and - 3A inhibited EMT and decreased cell invasiveness in cells with membrane E-cadherin. In cells with cytoplasmic E-cadherin, p120-1A stimulated EMT and cell invasiveness, while p120-3A prevented EMT and decreased cell invasiveness.	[72]
				**p120-3A**		
	Mutually exclusive exon	FGFR	Fibroblast Growth Factor Receptor	**FGFR lllc**	Increased level of FGFR-IIIc by mutually exclusive exon9 has been detected in a variety of tumors and correlated with tumor progression, such as increased grading and invasiveness, by promoting cancer cells to acquire mesenchymal characteristics.	[73,74,75]
	Exon skipping	RON	Macrophage Stimulating 1 Receptor	**ΔRON**	The skipping of Exon 11(ΔRON) brought about the deletion of an extra cellular domain that affects the proteolytic maturation of protein and increases cancer invasiveness through sustaining constitutively active status.	[76,77]
**Proliferation**	Exon inclusion	RPS6KB1	Ribosomal Protein S6 Kinase B1	**RPS6KB1-2**	RPS6KB1-2 made by inclusion of three cassette exons 6a, 6b, and 6c, caused the shorter isoform to lack a portion of the kinase domain. RPS6KB1-2 has contributed to cell proliferation and tumor growth via mTORC1 and 4E-BP1 phosphorylation.	[78,79]
	Exon inclusion	NUMB	NUMB Endocytic Adaptor Protein	**NUMB-PRR(L)**	In lung cancer cells, RBM10 mutations identified that disrupt splicing regulation of NUMB (Exon 9 inclusion) which is a key target of RBM5, 6, and 10 in the control of cell proliferation, to correlate with cell growth.	[80]
	Exon inclusion	SYK	Spleen Associated Tyrosine Kinase	**SYK(L)**	SYK(L), which includes exon 9 compared to the shorter isoform (SYK(S)), stimulates cell survival and tumor malignancy in many cancers by driving expression of epidermal growth factor.	[56]
	Exon skipping	MDM2	E3 Ubiquitin-Protein Ligase Mdm2	**MDM2-A**	Normal type of MDM2 could bind to p53 and facilitate proteasomal degradation of p53 as an ubiquitin ligase. Four of the splice isoforms (MDM2-A, -B, -C, and -D) by exon skipping in human cancers lack part of the p53-binding domain. Spliced isoforms could not bind to p53, enhancing degradation of p53 and cell proliferation.	[81,82,83]
				**MDM2-B**		
				**MDM2-C**		
				**MDM2-D**		
**Angiogenesis**	Alternative 3′ SS usage	VEGF	Vascular Endothelial Growth Factor	**VEGFxxx**	VEGFxxx isoforms, produced by alternative 3′SS usage, were overexpressed in many cancers, and resulted in proangiogenic effects.	[84]
	Alternative 3′ SS usage	VEGF	Vascular Endothelial Growth Factor	**VEGF 165**	sVEGFR1-113, a truncated version of VEGFR1, lacks its transmembrane and tyrosine kinase domains due to intron 13 retention.VEGF165 is a pro-angiogenic factor made by 3′ SS usage. One study identified that sVEGFR1-113 is considered to be a natural antagonist of VEGFA and upregulated under the mechanism associating the VEGF165/SOX2/SRSF2 network in anti-angiogenic therapied squamous lung carcinoma cells.	[85,86]
	Intron retention	VEGFR	Vascular Endothelial Growth Factor Receptor 1	**sVEGFR1-113**		
**Dysregulated** **metabolism**	Mutually exclusive exon	PKM	Pyruvate Kinase M2	**PKM2**	PKM2 had mutually exclusive exons containing Exon 10 not Exon 9 and is ubiquitously expressed in tumors. Substituting PKM2 with PKM1 in the tumor decreases lactate production and increases oxidative phosphorylation. Therefore, tumor growth is repressed.	[87,88]
**Immune** **response**	Exon skipping	BRAF	B-Raf Proto-Oncogene, Serine/Threonine Kinase	**BRAF(V600E)**	BRAF(V600E) transcripts by exon skipping (exon 4–8) brought about in-frame deletion of the N-terminal RAS-binding domain, resulting in melanoma cell resistance which is insensitive to inhibitors such as drug (PLX4032).	[89]
	Exon skipping	CEACAM1	CEA Cell Adhesion Molecule 1	**CEACAM1(S)**	The short isoform of CEACAM1, CEACAM1 (S) upregulated in many cancer types. This variant enlarged secretory IgA production by B cells and was associated with poor prognosis and peritoneal dissemination in gastric cancer.	[90,91,92]
**Cell cycle**	Intron retention	CCND1	CyclinD1	**CyclinD1b**	The formation of the cyclin D1b variant was linked with intron 4 retention, also concerned with cell cycle progression and proliferation in various cancers, competing with the same target, CDK4 with Cyclin D1a.	[93,94]

**Table 3:** The list of miRNAs dysregulated in various cancer types in the last 5 years.

Cancer Type	MiRNA	Subclass	Superfamily	Target Gene	Reference
**Renal cancer**	hsa-miR-224-3p	DNA transposon	DNA transposon	*ST3GalIV*	[247]
**Skin cancer**	hsa-miR-340-5p	DNA transposon	TcMar-Mariner	*RhoA*	[248]
**Bladder cancer**	hsa-miR-374b-5p	LINE	L2	*ZEB2*	[249]
	hsa-miR-330-5p	SINE	MIR	*circFARSA*	[162]
	hsa-miR-612	SINE	MIR	*ME1*	[250]
**Cervical cancer**	hsa-miR-224-5p	DNA transposon	DNA transposon	*PTX3*	[255]
	hsa-miR-374b-5p	LINE	L2	*JAM2*	[256]
**Endometrial cancer**	hsa-miR-582-5p	LINE	CR1	*AKT3*	[251]
**Oral cancer**	hsa-miR-1290	DNA transposon	TcMar-Tigger	-	[257]
	hsa-miR-1246	LTR	ERVL-MaLR	*CCNG2*	[258]
**Pancreatic cancer**	hsa-miR-1271	LINE	L2	E-*cadherin*, *ZEB1*, *TWIST1*	[252]
	hsa-miR-224-5p	DNA transposon	DNA transposon	*TXNIP*	[259]
	hsa-miR-887-3p	LINE	L2	*STARD13*	[253]
**Ovarian cancer**	hsa-miR-552	LINE	L1	*PTEN*	[254]
